# Self-harm in people experiencing homelessness: investigation of incidence, characteristics and outcomes using data from the Multicentre Study of Self-Harm in England

**DOI:** 10.1192/bjo.2022.30

**Published:** 2022-03-23

**Authors:** Caroline Clements, Bushra Farooq, Keith Hawton, Galit Geulayov, Deborah Casey, Keith Waters, Jennifer Ness, Anita Patel, Ellen Townsend, Louis Appleby, Navneet Kapur

**Affiliations:** Centre for Mental Health and Safety, School of Health Sciences, Manchester Academic Health Science Centre, University of Manchester, UK; Centre for Mental Health and Safety, School of Health Sciences, Manchester Academic Health Science Centre, University of Manchester, UK; Centre for Suicide Research, Department of Psychiatry, University of Oxford, Warneford Hospital, Oxford, UK; Centre for Suicide Research, Department of Psychiatry, University of Oxford, Warneford Hospital, Oxford, UK; Centre for Suicide Research, Department of Psychiatry, University of Oxford, Warneford Hospital, Oxford, UK; Centre for Self-harm and Suicide Prevention Research, Derbyshire Healthcare NHS Foundation Trust, Derby, UK; Centre for Self-harm and Suicide Prevention Research, Derbyshire Healthcare NHS Foundation Trust, Derby, UK; Centre for Self-harm and Suicide Prevention Research, Derbyshire Healthcare NHS Foundation Trust, Derby, UK; Self-Harm Research Group, School of Psychology, University of Nottingham, UK; Centre for Mental Health and Safety, School of Health Sciences, Manchester Academic Health Science Centre, University of Manchester, UK; Centre for Mental Health and Safety, School of Health Sciences, Manchester Academic Health Science Centre, University of Manchester, UK; and Greater Manchester Mental Health NHS Foundation Trust, Manchester, UK; and National Institute for Health Research (NIHR) Greater Manchester Patient Safety Translational Research Centre, Manchester, UK

**Keywords:** Self-harm, homelessness, emergency department, epidemiology, suicide

## Abstract

**Background:**

People who experience homelessness are thought to be at high risk of suicide, but little is known about self-harm in this population.

**Aims:**

To examine characteristics and outcomes in people experiencing homelessness who presented to hospital following self-harm.

**Method:**

Data were collected via specialist assessments and/or hospital patient records from emergency departments in Manchester, Oxford and Derby, UK. Data were collected from 1 January 2000 to 31 December 2016, with mortality follow-up via data linkage with NHS Digital to 31 December 2019. Trend tests estimated change in self-harm over time; descriptive statistics described characteristics associated with self-harm. Twelve-month repetition and long-term mortality were analysed using Cox proportional hazards models and controlled for age and gender.

**Results:**

There were 4841 self-harm presentations by 3270 people identified as homeless during the study period. Presentations increased after 2010 (IRR = 1.09, 95% CI 1.04–1.14, *P* < 0.001). People who experienced homelessness were more often men, White, aged under 54 years, with a history of previous self-harm and contact with psychiatric services. Risk of repetition was higher than in domiciled people (HR = 2.05, 95% CI 1.94–2.17, *P* < 0.001), as were all-cause mortality (HR = 1.45, 95% CI 1.32–1.59. *P* < 0.001) and mortality due to accidental causes (HR = 2.93, 95% CI 2.41–3.57, *P* < 0.001).

**Conclusions:**

People who self-harm and experience homelessness have more complex needs and worse outcomes than those who are domiciled. Emergency department contact presents an opportunity to engage people experiencing homelessness with mental health, drug and alcohol, medical and housing services, as well as other sources of support.

Homelessness is a priority issue following increases across high-income countries.^1,2^ Estimates from 2019 suggested that 280 000 people experienced homelessness in England, with over 4000 people classed as unsheltered or ‘rough sleepers’ and over 88 000 families in temporary accommodation.^3,4^ People experiencing homelessness are a high-risk group, vulnerable to physical and mental health problems as well as often being victims of crime.^1,5^ They are 60 times more likely to present to emergency departments compared with the general population, with higher levels of substance use and multimorbidity, despite having a lower mean age at the time of the presentation.^6^ Work using the Danish Homeless Register found that life expectancy of people experiencing homelessness was 22 years lower among men and 17 years lower among women, compared with people in the general population, and 28% of deaths were recorded as being due to external causes, such as suicide or accidents.^7^ In work looking at people in contact with psychiatric services when they died by suicide, people experiencing homelessness were more likely to have substance use problems compared with people who were domiciled.^8^

Self-harm is associated with suicide and a study conducted at one hospital in England found that risk of suicide after self-harm in people experiencing homelessness was more than twice that in domiciled people.^9^ People experiencing homelessness seem to have high rates of self-harm,^9,10^ with age-standardised incidence rates in Ireland 30 times higher in people experiencing homelessness compared with domiciled people.^11^ Among emergency department presentations for self-harm, people identified as experiencing homelessness were more likely to be male, to have used cutting as the main method of self-harm and to be admitted to a psychiatric ward for aftercare, compared with their domiciled counterparts.^11^

Work looking at self-harm and suicide in people experiencing homelessness is typically focused on subgroups, such as military veterans or young people, or limited to easily identifiable participants, such as those registered with homeless shelters.^12^ More work is needed that includes a broader definition of homelessness, such as people staying with friends or relatives, often referred to as the ‘hidden homeless’. With homelessness increasing, contemporary information on people who self-harm and experience homelessness is needed. This study used a large multisite data-set with follow-up information on mortality to describe the characteristics and outcomes of people who experienced homelessness and attended hospital following self-harm. The specific aims were: to examine trends over time in presentations for self-harm by people experiencing homelessness; to describe the common characteristics of this group (i.e. demographics, self-harm methods, history of contact with psychiatric services, problems that precipitated self-harm and referrals to aftercare services); and to look at outcomes in terms of repetition of self-harm and mortality.

## Method

Data on self-harm from the Multicentre Study of Self-harm in England between 1 January 2000 and 31 December 2016 were used. The data cover three hospitals in Manchester, one in Oxford and one in Derby. Self-harm is defined as any act of intentional self-poisoning or self-injury irrespective of the degree of suicidal intent or other motivation.^13^

### Data collection and measures

**Fig. 1:**
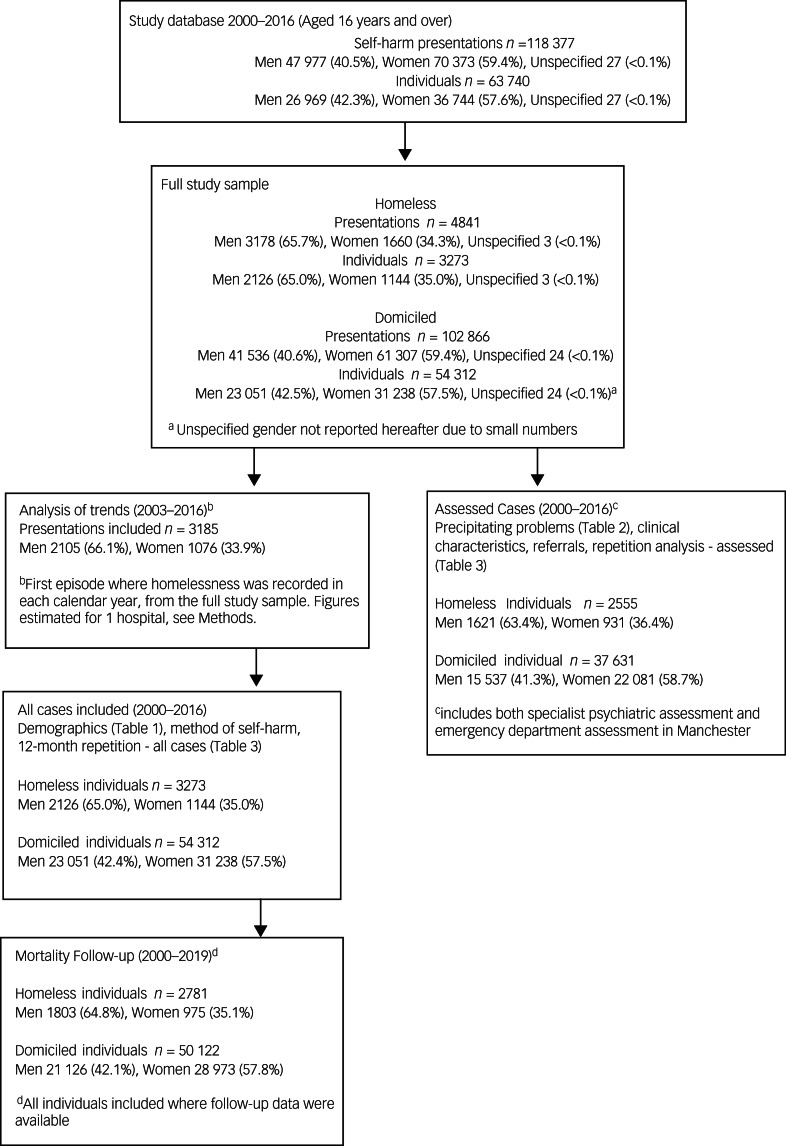
Flow chart describing overall numbers of cases included/excluded from statistical analyses.

Information on age, gender, ethnicity and method of self-harm was collected for all presentations via review of emergency department databases and clinical records. Further information was available for self-harm presentations where a specialist psychiatric assessment had taken place (or an assessment by emergency department clinicians in Manchester via completion of a study-specific form). Assessment data included: clinical characteristics (e.g. previous self-harm), historical or current contact with psychiatric services, problems that precipitated the self-harm and referrals for follow-up care. The absence of any specific reported problem or characteristic does not necessarily indicate it was not present, but that the patient or clinician did not identify it at the time of the assessment. Prior to 2003, data from Manchester were only available for individuals who received an assessment by a psychiatric specialist. Data on homeless status was not available from one hospital for 2015 and 2016. Fig. 1 gives details of included/excluded data.

### Case ascertainment of people experiencing homelessness

A broad definition of homelessness was used to ensure inclusion of the ‘hidden homeless’ (sometimes known as ‘sofa surfers’) as well as unsheltered people (e.g. ‘sleeping rough’) and people living in temporary or unsuitable accommodation, consistent with the definition of homelessness in English law.

Identification was based on (a) a person indicating they were homeless during an emergency department or specialist psychiatric assessment and/or (b) ‘no fixed abode’ stated in a patient's records. Only people over 16 years of age were included, as the experience of homelessness by the very young may be different from that of adults (31 children under 16 years were excluded from the analyses).^14^ People were assumed domiciled if they did not report homelessness and had a home address recorded in their patient record. The flow chart presented in Fig. 1 describes the cases included in the different analyses.

### Trends over time

Trends were based on the number of homeless individuals who presented in each calendar year (each person could only be counted once per year, to avoid bias due to frequent attenders). Data were restricted to 2003 onwards owing to missing information about non-assessed individuals in Manchester. Estimated figures were used for the hospital with missing homelessness information for 2015 and 2016, based on the overall proportion of homeless men and women who presented for self-harm in that hospital during 2014 (men: 3.9%; women: 0.9%) and applied to the total number of self-harm presentations to that hospital in 2015 (*n* = 634 men; *n* = 977 women) and 2016 (*n* = 695 men; *n* = 926 women). These estimates were included with the actual data from the rest of the hospital sites. A sensitivity analysis was run using only data from the hospitals with homelessness data across the whole study period to check that the estimate did not have any disproportionate effect on results.

### Characteristics

Descriptive statistics were based on individual-level analyses using a single ‘index’ episode. For people experiencing homelessness the index episode was the first episode on the study database where homelessness was recorded. For domiciled people the index episode was the first recorded episode of self-harm on the database. Individuals were allocated to one of two exclusive groups (homeless or domiciled) to ensure that people with both homeless and domiciled status recorded at different times during the study period were only included once.

### Repetition and mortality

Repetition of self-harm was based on any re-presentation to the study hospitals within 12 months of the index episode, using data from 2000–2015 to allow 12 months’ follow-up for all cases. All individuals were included in an age- and gender-adjusted model. A second multiple variable model included factors identified in previous research as being associated with repeated self-harm and suicide. These were: alcohol problems, drug problems, employment problems, age, gender, a history of previous self-harm, main method of self-harm (poisoning, self-injury, or poisoning and self-injury) and current receipt of psychiatric care.^6,11^ Analysis was restricted to assessed cases, as data on some covariates were dependent on receipt of an assessment (with a further sensitivity analysis run on all cases).

Data on deaths anywhere in the UK (except Northern Ireland) from any cause were supplied by NHS Digital (digital.nhs.uk) based on information from the Office for National Statistics (ONS). Individuals recorded on the multicentre database who presented between 2000 and 2016 were followed up to 31 December 2019, giving a maximum follow-up of 20 years (data were not available from one hospital for 2015 and 2016, but the follow-up period was consistent). Follow-up ended on the death of the individual or emigration outside of the UK. ONS suicide data included all individuals where a death had an underlying cause of intentional self-harm (i.e. ICD codes X60–84) or injury or poisoning of undetermined intent (i.e. Y10–34), as is usual practice in UK suicide research and official statistics.^15^ Accidental deaths (i.e. V01–X59) and deaths from all other causes (including natural causes) were also examined, as deaths due to these causes are known to be high in homeless populations.^16^ All individuals with follow-up data were included in an age- and gender-adjusted model. A second multiple variable model included the factors listed above as related to repetition of self-harm and suicide.

### Data analysis

All analysis were conducted using STATA/IC 14 for Windows.

#### Trends over time

Negative binomial regression models were used to test for changes in self-harm presentations over time, accounting for overdispersion in the data.

#### Characteristics

Pearson's chi-squared tests were used to compare characteristics, methods of self-harm and problems that precipitated self-harm between homeless and domiciled groups. Complete-case analysis was used to handle missing data, whereby only data on individuals with a valid (yes/no) response were included in analyses of each variable.

#### Repetition and mortality

Cox proportional hazards models were used to examine 12-month risk of repetition and mortality risk to 2019 in relation to homelessness. The proportion of overall mortality, mortality by suicide and undetermined causes, as well as mortality due to accidents and all other causes, were reported. Analyses run on the full sample were adjusted for age and gender. Gender-specific analyses were adjusted for age alone.

### Ethics

The monitoring systems in Oxford and Derby have approval from local research ethics committees to collect data on self-harm for local and multicentre projects. Self-harm monitoring in Manchester is part of a clinical audit system and has been ratified as such by the local research ethics committee. All three monitoring systems are fully compliant with the Data Protection Act 1998. All centres have approval under Section 251 of the National Health Service Act 2006 (formerly section 60 of the Health and Social Care Act 2001) to collect patient-identifiable information without patient consent and to release patient details to the Data Linkage and Extract Service of NHS Digital for the retrieval of mortality information on these individuals.

## Results

During the study period there were 4841 presentations to the study hospitals for self-harm where homelessness was recorded (4.5% of total presentations on the Multicentre Study database): 65.7% by men (*n* = 3178) and 34.3% by women (*n* = 1660). These presentations were made by 3270 individuals: 65.0% by men (*n* = 2126) and 35.0% by women (*n* = 1144). Information on gender was not available for three individuals. In comparison, the gender split in always domiciled individuals (*n* = 54 289) was 42.5% men (*n* = 23 051) and 57.5% women (*n* = 31 238). The proportions receiving a specialist biopsychosocial assessment were similar in homeless and domiciled people (57.6% *v*. 55.3%).

### Trends over time

**Fig. 2:**
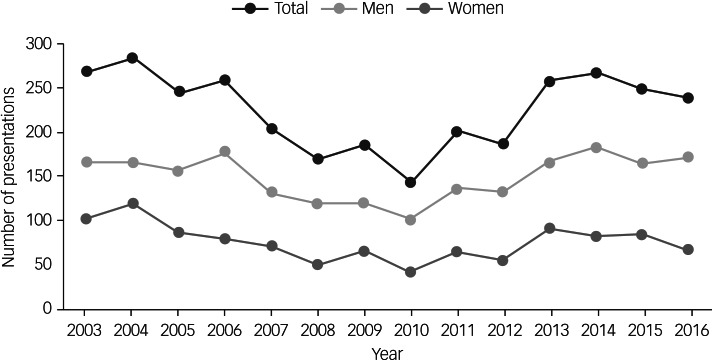
Number of self-harm presentations recorded each calendar year in individuals who were homeless, by gender. Data for one hospital were estimated for 2015 and 2016 based on the percentage of site-specific homeless presentations in 2014 and applied to the total number of presentations at that site in 2015 and 2016.

There was no change in the number of homeless individuals who presented to hospital for self-harm annually across the whole study period (IRR = 1.00, 95% CI 0.97–1.02, *P* = 0.78). This was the case for both men (IRR = 1.00, 95% CI 0.98–1.03, *P* = 0.66) and women (IRR = 0.98, 95% CI 0.95–1.01, *P* = 0.23). Sensitivity analysis with data from the hospital without homelessness status information for 2015 and 2016 removed showed little difference in results and no change in direction or significance for men or women. However, as Fig. 2 indicates, there appeared to be an initial decrease, followed by an increase in annual presentations after 2010 in both men and women. Additional analyses of these numbers during the 7 years before and after 2010 (2003–2009 and 2010–2016) showed an initial decrease in presentations of 8% per year prior to 2010 (IRR = 0.92, 95% CI 0.89–0.95, *P* < 0.001), followed by an increase of 9% each year between 2010 and 2016 (IRR = 1.09, 95% CI 1.04–1.14, *P* < 0.001).

### Demographic characteristics

Table 1 shows the demographic characteristics of the homeless and domiciled people. The homeless group was typically male, White and aged between 25 and 54 years. Experiencing homelessness was more likely in the Manchester area, especially among women. Women experiencing homelessness were younger than both domiciled women and homeless men, with 51.8% in the 16–24 year age group.

**Table 1:** Demographic profile of people experiencing homelessness and people who were domiciled who attended the emergency department following self-harm between 2000 and 2016^a^

Demographics	Homeless (*n* = 3273), *n* (valid %)^b^	Domiciled (*n* = 54 312), *n* (valid %)^b^	χ^2^ (d.f.), *P*
Male	2126 (65.0)	23 051 (42.5)	637.6 (1), <0.001
Female	1144 (35.0)	31 238 (57.5)	
Ethnicity			
White	2597 (90.8)	37 999 (87.9)	33.2 (3), <0.001
Black	57 (2.0)	1217 (2.8)	
South Asian	80 (2.8)	2097 (4.9)	
Other^c^	125 (4.4)	1935 (4.5)	
Age group			
16–24 years	1119 (34.4)	20 004 (37.4)	110.2 (3), <0.001
25–34 years	907 (27.9)	12 823 (23.9)	
35–54 years	1114 (34.3)	16 718 (31.2)	
≥55 years	111 (3.4)	4084 (7.6)	
Research site			
Manchester	2194 (67.0)	28 197 (51.9)	326.4 (1), <0.001
Oxford	650 (19.9)	12 563 (23.1)	
Derby	429 (13.1)	13 552 (25.0)	

a. Data are for 2000–2014 in one study site.

b. Only data on individuals with a valid (yes/no) response for the variable being assessed were included in each analysis.

c. Includes, for example, people from East Asia, the Middle East and North Africa.

### Method of self-harm

Self-poisoning was the most common method of self-harm in both groups. However, people experiencing homelessness more often used methods involving self-injury alone (27.4%, *n* = 898 *v*. 18.3%, *n* = 9945: χ^2^ = 168.2 (d.f. = 1), *P* < 0.001). This result held true in men (28.3%, *n* = 601 *v*. 23.5%, *n* = 5409: χ^2^ = 24.7 (d.f. = 1), *P* < 0.001) and women (26.0%, *n* = 297 *v*. 14.5%, *n* = 4531: χ^2^ = 114.2 (d.f. = 1), *P* < 0.001). Pure paracetamol was the most commonly used drug named in overdoses (33.2%, *n* = 749), followed by benzodiazepines (14.2%, *n* = 324) and selective serotonin reuptake inhibitor (SSRI) or serotonin and noradrenaline reuptake inhibitor (SNRI) antidepressants (14.0%, *n* = 317). However, the majority used other drugs (46.1%, *n* = 1060). Major tranquillisers and antipsychotics were more common in overdoses by people experiencing homelessness compared with those who were domiciled (8.8%, *n* = 201 *v*. 4.8%, *n* = 2072: χ^2^ = 72.35 (d.f. = 1), *P* < 0.001). Over half of presentations involved consumption of alcohol at the time of self-harm, but this did not differ from the domiciled group (57.7%, *n* = 1477 *v*. 56.9%, *n* = 22743: χ^2^ = 72.35 (d.f. = 1), *P* < 0.001).

### Clinical characteristics and referral from the emergency department

Previous self-harm was more common in people who experienced homelessness (80.9%, *n* = 1896 *v*. 49.9%, *n* = 16 820: χ^2^ = 844.3 (d.f. = 1), *P* < 0.001). A history of contact with psychiatric services was also more common in people who experienced homelessness (67.6%, *n* = 1546 *v*. 46.6%, *n* = 16 113: χ^2^ = 379.0 (d.f. = 1), *P* < 0.001), as was being in receipt of current out-patient psychiatric care (26.6%, *n* = 579 *v*. 17.0%, *n* = 5776: χ^2^ = 129.2 (d.f. = 1), *P* < 0.001). Referrals to in-patient psychiatric care (9.5%, *n* = 243 *v*. 6.3%, *n* = 2354: χ^2^ = 41.9 (d.f. = 1), *P* < 0.001), drug and alcohol services (11.4%, *n* = 290 *v*. 4.7%, *n* = 1750: χ^2^ = 222.9 (d.f. = 1), *P* < 0.001) and ‘other’ services, such as charity and third-sector organisations (15.2%, *n* = 388 *v*. 9.5%, *n* = 3562: χ^2^ = 88.3 (d.f. = 1), *P* < 0.001), were all more common in the group experiencing homelessness. Self-discharge from the emergency department was almost twice as common in people experiencing homelessness than in people who were domiciled; however, numbers were small overall (3.5%, *n* = 89 *v*. 1.9%, *n* = 705: χ^2^ = 32.0 (d.f. = 1), *P* < 0.001).

### Precipitating problems

Table 2 show the results of the analysis of the reported problems that precipitated self-harm. Relationship problems were less common in those experiencing homelessness (51.4%, *n* = 1313 *v*. 62.5%, *n* = 23 502: χ^2^ = 124.0 (d.f. = 1), *P* < 0.001). Alcohol, drug, and legal problems were more common in people experiencing homelessness, and housing problems were much more common, as may be expected (52.5%, *n* = 1260 *v*. 10.7%, *n* = 3865: χ^2^ = 3400.0 (d.f. = 1), *P* < 0.001). Furthermore, gender-separated analyses showed a similar pattern, with homeless men and women more commonly reporting legal, drug and alcohol problems, as well as more often reporting abuse as a precipitant. Comparing homeless men and women, homeless men more often reported alcohol (37.4%, *n* = 404 *v*. 27.6%, *n* = 152: χ^2^ = 15.7 (d.f. = 1), *P* < 0.001) and drugs (21.3%, *n* = 240 *v*. 15.8%, *n* = 89: χ^2^ = 7.5 (d.f. = 1), *P* = 0.006) as problems that precipitated self-harm, but abuse was much more common in homeless women (8.5%, *n* = 131 *v*. 17.7%, *n* = 151: χ^2^ = 43.2 (d.f. = 1), *P* < 0.001). Details of these comparisons are given in supplementary Table 1, available at https://doi.org/10.1192/bjo.2022.30.

**Table 2:** Problems reported as precipitating self-harm by people experiencing homelessness and people who were domiciled

Precipitants	Homeless (*n* = 2555), *n* (valid %)^a^	Domiciled (*n* = 37 631), *n* (valid %)^a^	χ^2^ (d.f.), *P*
Relationship problems	1313 (51.4)	23 502 (62.5)	124.0 (1), <0.001
Employment problems	480 (20.0)	7167 (19.9)	0.02 (1), 0.884
Financial problems	520 (21.7)	5340 (14.8)	81.8 (1), 0.001
Housing problems	1260 (52.5)	3865 (10.7)	3400.0 (1), <0.001
Legal problems	257 (10.7)	1640 (4.6)	182.0 (1), <0.001
Alcohol problems	556 (34.0)	5949 (21.4)	143.1 (1), <0.001
Drug problems	330 (19.5)	2157 (7.4)	313.5 (1), <0.001
Physical health problems	187 (7.8)	3906 (10.8)	21.8 (1), <0.001
Mental health problems	605 (25.2)	8656 (24.1)	1.8 (1), 0.184
Abuse^b^	282 (11.8)	2968 (8.3)	35.8 (1), <0.001

a. Only data on individuals with a valid (yes/no) response for the variable being assessed were included in each analysis.

b. Abuse includes any or a combination of physical, emotional and sexual abuse.

### Repetition and mortality

Full results of the repetition and mortality analyses are shown in Table 3. More homeless than domiciled people repeated self-harm within 12 months (HR = 2.48, 95% CI 2.31–2.65, *P* < 0.001). When restricting the analysis to assessed cases (HR = 2.53, 95% CI 2.34–2.73, *P* < 0.001), the inclusion of additional explanatory variables (i.e. alcohol, drug and employment problems, age, gender, a history of previous self-harm, method of self-harm and current receipt of psychiatric care) decreased the hazard ratio but it remained significant (HR = 2.07, 95% CI 1.86–2.30, *P* < 0.001). The same pattern was seen in gender-specific comparisons.

**Table 3:** Cox proportional hazards models for 12-month repetition and mortality follow-up, for people experiencing homelessness and domiciled people

	Twelve-month repetition of self-harm^a^			Mortality follow-up		
	Homeless (*n* = 3110), *n* (%)	Domiciled (*n* = 46 458), *n* (%)	HR (95% CI)	Homeless *n* = 2781 *n* (%)	Domiciled *n* = 50 122 *n* (%)	OR (95% CI)
All cases	928 (29.8)	7109 (13.8)	2.48 (2.31−2.65)*			
Assessed cases^b^	755 (30.7)	4970 (13.8)	2.53 (2.34−2.73)*			
Assessed cases (including explanatory variables)^b^	755 (30.7)	4970 (13.8)	2.07 (1.86−2.30)*			
Total mortality				481 (17.3)	5595 (11.2)	1.44 (1.31−1.59)*
Total mortality (including explanatory variables)				481 (17.3)	5595 (11.2)	1.20 (1.02−1.42)*
Suicide or undetermined intent				65 (2.4)	803 (1.6)	1.25 (0.97−1.61)
Accidental causes				120 (4.4)	647 (1.3)	2.93 (2.41−3.57)*
All other causes^c^				274 (9.9)	3985 (8.0)	1.19 (1.06−1.35)*

a. Presentations from 2016 were excluded to give 1-year follow-up for all cases.

b. The denominator was restricted to assessed cases: 2555 homeless cases, 37 631 domiciled cases.

c. Includes ICD F00–F99 mental and behavioural, R99 ill-defined or unspecified, all natural causes.

* Significant at *P* < 0.05.

Mortality data were available for 2781 (85.0%) people experiencing homelessness and 50 122 (92.3%) domiciled people. The incidence of all-cause mortality was higher in people who were homeless than in those who were domiciled (17.3%, *n* = 481 *v*. 11.2%, *n* = 5595: HR = 1.45, 95% CI 1.32–1.59, *P* < 0.001). This difference was maintained when additional factors were entered into the model (HR = 1.20, 95% CI 1.02–1.42, *P* = 0.029). Suicide and death due to undetermined causes did not differ between groups (HR = 1.25, 95% CI 0.97–1.61, *P* = 0.088). People experiencing homelessness were nearly three times more likely to die due to accidental causes (4.4%, *n* = 120 *v*. 1.3%, *n* = 647: HR = 2.93, 95% CI 2.41–3.57, *P* < 0.001).

## Discussion

This study examined trends, characteristics and mortality outcomes in self-harm by people experiencing homelessness using detailed multisite clinical data. People recorded as experiencing homelessness were predominantly men aged between 25 and 54 years. Although no overall trends were detected across the whole of the study period, an initial decrease in self-harm presentations early in the study, followed by a rapid increase after 2010 was found. Previous self-harm was reported by 81% of people experiencing homelessness, and often occurred alongside current or previous contact with psychiatric services, as well as problems with substance misuse, finances, legal issues and abuse. Homelessness was associated with a doubled risk of repetition of self-harm within 12 months and higher all-cause mortality than domiciled status, especially in deaths due to accidental causes, which were three times more common in the homeless group.

Although demographic characteristics were broadly consistent with previous work, 12-month repetition of self-harm was higher than found in a recent Irish study.^11^ Mortality analysis showed no difference in risk of death due to suicide between homeless and domiciled people who self-harm.^7^ Accidental deaths, however, were much more common in the homeless group, which is in line with previous studies.^5,17^ These deaths may be misclassified suicides, particularly in relation to overdose, and the current results may therefore be an underestimate of suicide in this population.^5,6^ An excess in overall mortality, including from natural causes, is consistent with older work that identified an excess of physical health problems in people experiencing homelessness.^6,17^ However, physical health problems were not commonly reported as a factor precipitating self-harm in this study and it may be that those experiencing homelessness are less likely to perceive physical health problems as being a principal concern in relation to self-harm, consistent with reports that housing and alcohol are more critical in this population.^6,17^

No previous work was identified that looked at changes over time in self-harm presentations by individuals experiencing homelessness. Increased presentations after 2010 are consistent with increases in homelessness following the economic recession. This was possibly driven by impacts of the financial crisis, such as increases in unemployment, benefit cuts, financial instability and changes in the availability of support services due to austerity measures, and mirrors an increase in self-harm more generally.^18,19^ Owing to a lack of reliable data on the homeless population in England (i.e. the denominator) it was not possible to calculate population-based rates of self-harm, and therefore the results may reflect increases in the homeless population rather than increases in self-harm itself.

### Strengths and limitations

This study used a large, detailed sample of people who presented to hospital following self-harm and who were experiencing homelessness, with data spanning a 20-year study period. Additional strengths of the study are that it was possible to include different types of homelessness, such as people staying with relatives or friends, as well as the long follow-up period for mortality outcomes. One limitation of this study is that it is based on hospital presentations in predominantly urban areas and results may not therefore be applicable to self-harm in other settings. However, the multisite data represent areas with different demographic and economic profiles, including areas of high and low deprivation, and may therefore be generalisable to England as a whole. Although few similar studies were identified in the international literature, the general characteristics of people who experience homelessness and self-harm were in line with findings from other countries.^5,11,17^

It was not possible to verify the status of those identified as ‘homeless’ in this work. We opted to define homelessness as broadly as possible, as awareness of the hidden homeless and those in temporary accommodation has increased in recent years.^1^ The social exclusion of people who are homeless means that it is difficult to collect data on them. Although there may be limitations to the current work it is important that this population is treated equitably, which includes being the focus of research studies despite the challenges of reliable case identification.^6,20^ It is also possible that some unassessed people experiencing homelessness were incorrectly categorised and placed in the domiciled group, which would inflate the apparent proportion of people experiencing homelessness who received an assessment – this may be particularly true of the ‘hidden homeless’, who may give the address where they are staying and would therefore have a valid postcode, which was part of the domiciled ascertainment criteria.

Some analyses were conducted on a subsample, as receipt of an assessment determined the availability of information about many factors. Information was not available on all factors that may be relevant to self-harm in people who experience homelessness. For example, details of psychiatric diagnoses, which are known to be prevalent in people who are homeless and also to increase risk of self-harm repetition, were not available.^7^ Other factors, such as duration of homelessness and adherence to psychiatric treatment, were unknown. Additional important factors such as these would be worth investigating in future studies via data linkage to mental health and/or primary care records, along with qualitative studies to develop further understanding of the factors and processes that contribute to self-harm in this population.

### Clinical implications

Issues involving drugs and alcohol were particularly pronounced in the homeless group and more so in men than in women, but referrals to drug and alcohol services were low, at around 10%.^11^ Substance use may be a coping mechanism for some experiencing homelessness and cessation without resolution of housing issues may not be wanted.^20^ Harm-reduction and safer-drinking strategies have been shown to be a more helpful and acceptable approach in some studies.^21^ Closer monitoring by mental health services may help to address changes in illness severity and reduce risk of repetition of self-harm and suicide, but may be challenging in this population. Social prescribing of support tailored to the needs of people experiencing homelessness, such as peer-run well-being groups, has shown positive results.^22^ There is evidence that increasing social capital (i.e. networks of relationships between people who live in a society) via social prescribing and one-to-one support from outreach workers improves health outcomes and engagement with services^22,23^ Previous work showed that men in mid-life who self-harm were less likely to be in contact with mental health services than women of the same age who self-harm, but service and engagement initiatives directed towards men in mid-life may not be appropriate for those also experiencing homelessness and therefore adaptations may be required.^19,24^

Those experiencing homelessness were more often referred to in-patient psychiatric care than their domiciled counterparts. In-patient admission may indicate higher severity of mental health problems, but referrals may also be made to meet the need for a safe place to stay (particularly in relation to those who are unsheltered).^25^ However, a recent Danish study showed that psychiatric in-patients were at increased risk of becoming homeless following discharge, especially those with substance use problems or previous experiences of homelessness.^26^ Initiatives such as Housing First, which brings together a range of support services to promote establishing stable housing arrangements as well as improved mental health, may be more appropriate and have shown some success in reducing in-patient care and emergency department contacts among people who are homeless.^27,28^

This study showed that people experiencing homelessness who attend hospital for self-harm are at greater risk of repetition of self-harm and mortality due to accidental causes than domiciled people who self-harm. A high frequency of previous self-harm, along with current and/or previous contact with psychiatric services and drug and alcohol use, suggest a group with complex needs. These results will remain important in the post-COVID-19 period, as contacts with mental health services decreased during the pandemic and long-term economic effects remain unclear.^29^ The point of contact created via presentation to the emergency department following self-harm may therefore be an important route to accessing help and it is important that appropriate aftercare is accessible. A biopsychosocial assessment in line with National Institute for Health and Care Excellence (NICE) guidance is essential for anyone who attends the emergency department following self-harm, and questions about living arrangements and the potential for future homelessness should be included.^30^ Brief interventions such as safety planning could be incorporated into assessments and may help reduce repetition of self-harm.^31^ Multi-agency approaches, with referrals to drug and alcohol services, support for improving health and improved access to physical health services, as well as social and housing services, may benefit this high-risk group and reduce premature mortality, suicide and self-harm. Indeed, recent NHS initiatives using one-to-one outreach workers, supported by multidisciplinary teams, to tackle housing, mental and physical health problems as well as drug and alcohol use are promising but need further evaluation.^32^ Integrated co-located services and homeless shelters coordinating care have also been proposed as helpful for initiation and engagement with physical and mental health services, but crucial to reducing risk of self-harm in this population is support to access safe and stable long-term housing.^33^

## Data Availability

The data in this study are not publicly available because they contain information that could compromise the privacy of research participants. The corresponding author can be contacted for further details.

## Supplementary material

For supplementary material accompanying this paper visit http://doi.org/10.1192/bjo.2022.30. click here to view supplementary material
