# Supplementary material for: Self-harm in people experiencing homelessness: investigation of incidence, characteristics and outcomes using data from the Multicentre Study of Self-Harm in England
Source: BJPsych Open. 2022 Mar 23;8(2):e74. doi: 10.1192/bjo.2022.30 (PMC9059614; doi:10.1192/bjo.2022.30)
Supplement: Supplementary file 1 [file S2056472422000308sup001.docx]

**Supplementary Table S1:** Gender split and gender comparison analysis of problems that precipitated self-harm for homeless men and homeless women (only assessed cases included in analysis).

| **Precipitants** | **Homeless Men**  **n=1,621 (valid%)** | **Domiciled Men**  **n=15,537**  **(valid%)** | **X2 (d.f) p** | **Homeless Women**  **n=931**  **(valid%)** | **Domiciled Women**  **n=22,081**  **(valid%)** | **X2 (d.f) p** | **Homeless men vs. homeless women**  **X2 (d.f) p** |
| --- | --- | --- | --- | --- | --- | --- | --- |
| Relationship problems | 781 (48.2) | 9,178 (59.1) | 71.5 (1) p<0.001 | 531 (57.1) | 14,316 (64.8) | 23.7 (1) p<0.001 | 18.6 (1) p<0.001 |
| Employment problems | 348 (22.7) | 3,471 (23.3) | 0.32 (1) p<0.570 | 132 (15.4) | 3,693 (17.5) | 2.6 (1) p<0.107 | 18.4 (1) p<0.001 |
| Financial problems | 369 (24.0) | 2,688 (18.1) | 32.7 (1) p<0.001 | 151 (17.6) | 2,652 (12.6) | 18.6 (1) p<0.001 | 13.6 (1) p<0.001 |
| Housing problems | 868 (56.5) | 1,798 (12.1) | 2000.0 (1) p<0.001 | 392 (45.6) | 2,066 (9.8) | 1100(1) p<0.001 | 26.2 (1) p<0.001 |
| Legal problems | 183 (11.9) | 1,008 (6.8) | 54.8 (1) p<0.001 | 74 (8.6) | 632 (3.0) | 83.8 (1) p<0.001 | 6.3 (1) p=0.012 |
| Alcohol problems | 404 (37.4) | 3,119 (26.9) | 54.6 (1) p<0.001 | 152 (27.6) | 2,827 (17.5) | 37.3 (1) p<0.001 | 15.7 (1) p<0.001 |
| Drug problems | 240 (21.3) | 1,365 (11.3) | 97.0 (1) p<0.001 | 89 (15.8) | 792 (4.7) | 140.8 (1) p<0.001 | 7.5 (1) p=0.006 |
| Physical health problems | 122 (8.0) | 1,728 (11.6) | 18.6 (1) p<0.001 | 64 (7.4) | 2,176 (10.3) | 7.4 (1) p=0.007 | 0.20 (1) p=0.657 |
| Mental health problems | 385 (25.1) | 3,550 (23.8) | 01.1 (1) p=0.0.287 | 220 (25.6) | 5,101 (24.1) | 1.0 (1) p=0.329 | 0.1 (1) p=0.773 |
| Abuse | 131 (8.5) | 746 (5.0) | 34.1 (1) p<0.001 | 151 (17.7) | 2,221 (10.5) | 42.4 (1) p<0.001 | 43.2 (1) p<0.001 |
|  | | | | | | | |
